# Assessment of Lexicographic Minimax Allocations of Blue and Green Water Footprints in the Yangtze River Economic Belt Based on Land, Population, and Economy

**DOI:** 10.3390/ijerph16040643

**Published:** 2019-02-21

**Authors:** Gang Liu, Fan Hu, Yixin Wang, Huimin Wang

**Affiliations:** 1 State Key Laboratory of Hydrology of Water Resources and Hydraulic Engineering, Hohai University, Nanjing 210098, China; 2 Institute of Management Science, Hohai University, Nanjing 210098, China; 18551673413@163.com (F.H.); yxwang1135@hotmail.com (Y.W.); 3 Hohai University Coastal Development and Protection Collaborative Innovation Center, Nanjing 210098, China

**Keywords:** water footprint, lexicographic minimax allocation, Gini coefficient, Theil entropy

## Abstract

To assess different impacts of land, population and economy factors on the lexicographic minimax optimal allocation of blue and green water footprints, a comprehensive discriminant rule is constructed in this paper based on the Gini coefficient and Theil entropy index. The proposed rule is employed to estimate the influence of the aforesaid factors (land, population and economy) on the corresponding allocation schemes from a fairness perspective. To demonstrate its applicability, the proposed approach is applied to a water resources allocation study for 11 provinces in the Yangtze River Economic Belt (YREB). The results indicate that: (1) the economy-based lexicographic allocation of water footprints (LAWF) is more equalitarian for the provinces with high water footprint quotas. The land area-based LAWF is more equalitarian for the provinces with low water footprint quotas. The population-based LAWF is more equalitarian for the provinces with medium water footprint quotas. (2) The contribution of intra-regional variation in the population-based LAWF scheme is the largest of the three schemes. The inter-regional variation contributed the largest in the land area-based LAWF scheme. (3) Two synthetic schemes which integrate multiple factors among land area, economy and population are more equalitarian than the three single-factor schemes. Compared with the original situation which is an equalitarian but ineffective allocation, the two synthetic schemes have greater effect on the improvement of the supply-demand balance of water resources carrying capacity. Therefore, the defect of the population, economy and land area factors acting alone should be resolved by designing a weighting system, in order to optimize the allocation of water resources.

## 1. Introduction

With continuing growth of population, the shortage of water resources has increasingly become a critical restraining factor of environmental and social sustainable development all over the world. Especially in human high-intensity activity areas, secondary disasters such as water pollution, groundwater over-exploitation and seawater intrusion caused by water shortage have become a major crisis affecting the sustainable development of public health in the areas. China is also faced with a conflict between the severe shortage of water resources and the rapid development of its economy and society. The annual water shortage in China is as high as 50 billion m^3^, and China is listed by the United Nations as one of the 13 countries with the greatest water scarcity. To make things even worse, it is notorious that China has serious spatially and temporally imbalanced distribution of water resources [1]. As an important grain producing area and an economic fortress with a dense population, the Yangtze River Economic Belt (YREB) in China accounted for over 40% of the water consumption and economic output of the country in 2014. But allocation of water resources in the YREB faces even more difficulties. On the one hand, the spatial and temporal distribution of the water resources is extremely uneven in the YREB: the highest annual rainfall in a YREB province can be as high as 4.8 times the lowest annual rainfall in another province in the area and the maximum variation of annual rainfall in the same YREB province across different years can reach a ratio of 2.5 times. On the other hand, the water resources per capita and water resources per unit land area in the YREB are scarce and poorly distributed. The amount of water resources per unit area of arable land in the YREB is approximately 70% of the world average, while the amount of water resources per capita in the YREB stands at 30% of the world average. In addition, the proportion of industrial, agricultural and domestic water consumption in the YREB is 31.7%, 53.4% and 13.8% respectively. The water use efficiency of industrial sectors in the YREB is low. The average water consumption per unit of industrial added value in 2014 is 24.9% higher than the national average. If agriculture and service industries are included, the water consumption per unit GDP in the YREB is 4.6% lower than the national average. Water conflict has become a key bottleneck restricting environmental and social sustainable development of the YREB.

To address the conflict between ecological protection and economic development, the Chinese government promulgated “Outline of Yangtze River Economic Belt Development Plan” in 2016 to elaborate its development strategy of the YREB. This outline clearly identifies the first development priority as the protection and restoration of ecological environment in the YREB. It proposes the concept of green development with water resources as the core concern for the first time. The outline hopes to rely on efficient allocation of water resources in the area to promote optimization and reorganization of development factors such as population, economy, and land to ensure environmental and social sustainable development of the YREB. The key to implementing this green development strategy is to analyze the actual demand of water resources in different provinces from a water footprint perspective and, then, improve water demand management through optimal allocation of water footprints to promote water use efficiency and protect water, economic, and social security in the YREB.

The concept of water footprints takes a holistic view on water resources [2]. It accounts for water resources needed for all products and services consumed by a known population (a country, a region, or a person) for a certain period of time. It can be defined from several major dimensions of products, consumer groups, vendors, specific geographic regions, and residents. To achieve an equitable allocation of water footprints in the YREB, we adopt a lexicographic minimax algorithm. Since the lexicographic minimax optimal allocation scheme significantly depends on the influencing factor adopted in the algorithm, it is necessary to establish a proper discriminant rule to assess different allocation results based on different factors such as land, population, and economy.

## 2. Literature Review

Since the British scholar Allan [3], proposed the concept of virtual water in 1993, and the Dutch scholar Hoekstra [2] introduced the water footprint concept in 2002, research on water footprints has mainly concentrated in three areas: theoretic framework and quantitative methods, case studies, and water resources evaluation and management research. For instance, Feng et al. [4] conducted a spatial analysis of the water footprint in Britain. Hoekstra [5] examined the relationship between water footprints and supply chains. Egan [6] proposed a water footprint quantification and evaluation framework. Naranjo-Merino et al. [7] assessed regional green and blue water footprints of cocoa production in Norte de Santander, Colombia. Mekonnen and Hoekstra [8] constructed a dynamic moisture balance model based on a grid scale, thereby quantifying water footprints of global crops. Ewing et al. [9] integrated ecological and water footprints into a “footprint family”. Hoekstra et al. [10] attempted to evaluate water footprints around the globe. Zhang et al. [11] suggested that the notion of water footprints provides a new way to clarify the impact of human production and consumption activities on water resources. By evaluating water footprints, management authorities can achieve sustainable utilization of water resources. Along this line, Ercin and Hoekstra [12] analyzed the changes in global consumption and production of water footprints for future generations as well as economic development and changes in production and consumption structures. Their research furnishes a valuable reference for governments and relevant authorities to develop proper water resources management strategies.

As commonly recognized by the academic community, allocation of water resources should follow the principles of fairness, efficiency, sustainability. Many scholars have studied allocation equity. For instance, Ogryczak [13] proposed that the conditional mean criterion can guarantee fair distribution of resources and further showed that equitable preferences can be modeled by properly selecting weights in an Ordered Weighted Averaging (OWA) aggregation method. With a finite number of entities, the OWA aggregation function can be approximated by a lexicographic ranking [14] for the ordered output value. In multi-objective decision making, the lexicographic minimax algorithm is a useful technique of seeking Pareto optimal solution based on fairness. The lexicographic minimax algorithm is proved efficient thanks to the influencing factor *α_i_* compared to the traditional equal proportion configuration and allocation method [15].

The influencing factor in the lexicographic minimax algorithm is the key to guaranteeing fairness, efficiency and sustainability. The methods to judge the validity of the influencing factors mainly include the Gini coefficient, Theil index, information entropy, among others. The Gini coefficient and the Theil index have been popular research topics in recent years. The Gini coefficient evolves from an unequal index proposed by the Italian economist Gini [16] and is an internationally recognized classic indicator of overall income gaps. Chen [17] further noted that it is essentially a statistical indicator of the dispersion of discrete variables. Some scholars have introduced the Gini coefficient into the research of resource allocation and fairness evaluation. Along this line, Zhou et al. [18] applied the environmental Gini coefficient model to evaluate the inequality of water governance responsibility allocation. Malakar et al. [19] used the Gini coefficient to estimate the overall inequality in water supply in India. Based on the Gini coefficient, Seekell [20] quantitatively assessed the effect of international trade on equality in global virtual water distributions. The Theil Entropy, first proposed by the Dutch economist Theil [21] in 1967, has the property of symmetry and scale invariance, and it also satisfies the Pigou—Dalton Criterion, which measures the contributions of intra-group and the inter-group variations to the total variation in an imbalanced data set. It is one of the most important methods for measuring distributions of discrete and imbalanced data and is widely used in the fields of regional economy [22], income distribution [23] and resource allocation [24].

In summary, the notion of water footprints arises as a popular research topic in water resources management, and research results have demonstrated that water footprints can be a reasonable and practical index to allocate water resources. Existing studies have shown that the lexicographic minimax algorithm can optimize resource allocation. As far as the authors are aware, limited studies have been carried out to assess the mechanism and impact of different influencing factors on the lexicographic optimal allocation of water footprints (LAWF). By applying the LAWF to the YREB, this paper puts forward a comprehensive discriminant approach based on the Gini coefficient and Theil entropy to analyze the performance of LAWF based on three different influencing factors, land, population, and economy. Due to lack of data, water footprints in this article are confined to blue and green water footprints. By adopting the proposed discriminant rule, we systematically analyze the allocation mechanism and impact of the three factors, land, population, and economy, on the corresponding allocation scheme.

## 3. Materials and Methods

### 3.1. Region Under Investigation

**Figure 1:**
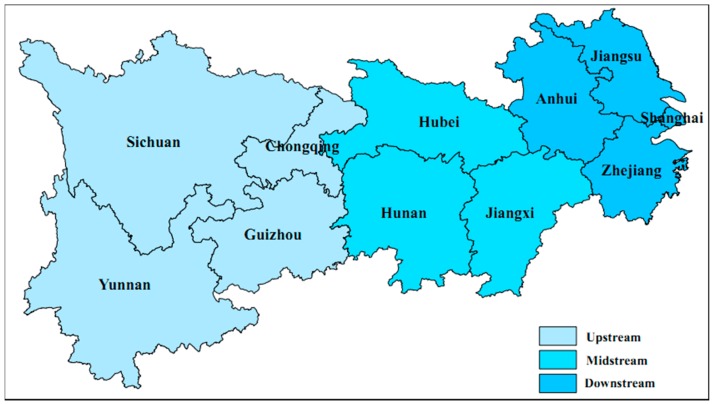
The YREB area in China [25].

As China’s major economic powerhouse and commodity production base, the YREB has been identified as one of its key regional economic development strategies. Geographically, the YREB consists of nine provinces and two provincial-level municipalities in the Yangtze River Basin, extending from Shanghai in the east to Yunnan in the west. It covers a territory of approximately 2.05 million km^2^ and approximately 40% of the nation’s population resides in this area. Figure 1 shows a sketch of the geographic location of the YREB [25].

Traditionally, the YREB is divided into three regions: upstream, midstream, and downstream, as properly colour-coded in Figure 1. The upstream region consists of three provinces, Sichuan, Guizhou, and Yunnan, and a provincial-level municipality, Chongqing. The midstream region includes three provinces, Hubei, Hunan, and Jiangxi. The downstream region refers to three provinces, Jiangsu, Zhejiang, Anhui, and a provincial-level municipality, Shanghai. Without causing confusion, we shall hereafter refer to these 11 YREB jurisdictions as provinces.

### 3.2. Data Collection

In 2013, “Relying on the golden waterway to build the Yangtze River Economic Belt” was written into the work report of the Chinese government. This year’s state can be used as a benchmark value to reflect the changes before and after the transformation of the development concept of Yangtze River Basin. In this paper, the 11 YREB provinces are selected as the research area, and the raw data of water resources and other economic and social statistics in 2013 are collected and analyzed. The main sources of raw data are taken from the *China Statistical Yearbook (2014)*, the *Statistical Yearbook (2014)* of the 11 provinces, the *China Environmental Statistical Yearbook (2014)*, the *China Water Resources Bulletin (2013)*, and the *Yangtze River Basin and Southwest River Water Resources Bulletin (2013)*. The meteorological data are drawn from the China Meteorological Data Network (http://data.cma.cn/), and the crop coefficient data are gathered from the Food and Agriculture Organization (FAO) World Weather Database (http://www.fao.org). The cultivated crops in this area mainly consist of paddy rice, wheat, corn, sorghum, beans, potato, peanut, rapeseed, tobacco, vegetables, fruits among others, and livestock products including beef, lamb, pork, chicken, milk, eggs, and more. Table 1 below shows the basic data on land area, population, GDP, available and consumed water resources in the 11 YREB provinces in 2013. It should be noted that a significant portion of available water resources listed in Table 1 is not ready for human use as it is often located in remote mountainous areas beyond human reach. Additional data for blue and green water footprint accounting are given in Appendix B.

### 3.3. Models

This paper first evaluates the current water demand structure in the 11 YREB provinces from a water footprint perspective by decomposing water consumption into the following four categories: agriculture, industry, living, and ecology. Second, by using land area, population, and the GDP (as a proxy of economy), respectively, as the influence factor, we obtain different allocation schemes of water resources in the 11 YREB provinces by applying a lexicographic minimax algorithm [25]. We then construct a comprehensive index family to assess the three aforesaid allocation schemes. By using the data of the 11 YREB provinces, the proposed discriminant framework is applied to evaluate the mechanism and influence validity of different LAWF allocation schemes.

#### 3.3.1. Model of LAWF Considering Influencing Factors

Generally speaking, water footprints include blue, green, and grey portions. However, due to limited data available for determining grey water footprints, existing studies typically omit this part when water footprints are considered for water resources management in China [26,27,28]. Therefore, this paper follows suit and considers blue and green water footprints only. Without causing confusion, water footprints hereafter refer to blue and green water footprints.

It is assumed that the water footprint for each province in YREB is *x_i_*, 0 ≤ *x_i_* ≤ *WF_i_*, *i* = 1, 2, …, *n*, where *WF_i_* is the upper bound of the water footprint in province *i* given by the original water footprints. The water footprint consists of agricultural, industrial, domestic and ecological water footprints. First, denoting the agricultural water footprints by *WF_ai_*, it can be expressed as:

(1) $$WF_{ai}=\sum_{j}\frac{10\cdot K_{ij}\cdot \sum_{d}ET_{ij}(d)\cdot S_{ij}}{g_{ij}}+\sum_{k}M_{ik}\cdot \frac{\int_{birth}^{slaughter}\left\{water_{ikd}+water_{ik,serve}+water_{ika}+\sum_{h}SWD(h)\times C_{ik}(h)\right\}dt}{weight_{ika}}$$

In this model, subscripts *i*, *j*, and *k* refer to province *i*, crop *j*, and animal *k*, respectively. *K_ij_* is the average crop coefficient during the cultivation period. As no region-specific data for the area under study, the FAO recommended values are employed in our model calibration. *ET_ij_* (mm/day) is the daily evapotranspiration of crop *j*. The factor 10 converts mm into m^3^/km^2^ and the inner summation accounts for the total evapotranspiration from day 1 to the final day of the cultivation period [29]. *g_ij_* (ton/km^2^) is the unit output of crop *j*. *S_ij_* (ton/year) is the total annual output of crop *j*. *M_ik_* (ton/year) is the annual output of animal *k* in province *i*. *water_ikd_* (m^3^/day) indicates the daily amount of water consumed by animal *k*, and *water_ik,serve_* (m^3^/d) represents the total volume of water used to clean the farmyard and the animal as well as any other services necessary to maintain the environment during the entire life span of animal *k*. The amount of water consumed by an animal from the fodder during its life span consists of two parts: *water_ika_* (m^3^/d) stands for the actual water required for preparing the feed while the second part is the virtual water incorporated into various fodder components. *SWD*(*h*) (m^3^/ton) denotes water demand of feed crop *h*, *C_ik_*(*h*) (ton/day) signifies the quantity of feed crop *h* consumed by animal *k*. *weight_ika_* (ton) is the live weight of animal *k* at the end of its life span.

Second, denoting the agricultural water footprints by *WF_ni_*, it can be expressed as:

(2) $$WF_{ni}=Q_{iMI}\cdot P_{iIO}+VW_{iI}-VW_{iO}+\sum_{g}U_{ig}\cdot P_{ig}+A_{i}\cdot \eta_{i}\cdot EP_{i}$$

In this model, *Q_iMI_* (m^3^/RMB) is the water consumption per ten thousand yuan of Renminbi (the Chinese currency) of industrial product, and *P_iIO_* (RMB/year) is the gross industrial product in ten thousand of RMB. *VW_iI_* (m^3^/year) is the virtual water amount due to inter-provincial import trade and *VW_iO_* (m^3^/year) is the virtual water amount owing to inter-provincial export trade. *U_ig_* (m^3^/person) is the per capita water quota; *P_ig_* (person/year) is the annual population; *A_i_* (km^2^) is the urban area; *η_i_* is the urban greening coverage excluding water surface; and *EP_i_* (m^3^/km^2^.year) is the annual evapotranspiration of plants per unit area.

Additionally, based on the relevant research results at home and abroad, the land area, the population and the economic output are taken as the influencing factors. For the optimal allocation of the blue and green water footprints with limited configuration, the maximum value of the Lexicographic minimization is defined as follows:

(3) $$\begin{array}{l} Lex\;\underset{x}{\min}[\alpha_{i}\cdot f(x_{i})]=Lex\;\underset{x}{\min}[\alpha_{i}\cdot \frac{WF_{i}-x_{i}}{WF_{i}}]\\ s.t.\left\{ \begin{array}{l} WF_{i}=WF_{ai}+WF_{ni}\\ \alpha_{i}=\{\frac{\omega_{ti}}{\sum_{i}\omega_{ti}}|0<\alpha_{i}<1\}\\ \sum_{i\in N}\alpha_{i}\cdot x_{i}\leq \sum_{i\in N}Q_{i}\\ {\underline{Q}}_{i}\leq x_{i}\leq {\bar{Q}}_{i}\forall i\in N \end{array}\right. \end{array}$$

Then $f(x_{i})=\frac{WF_{i}-x_{i}}{WF_{i}}$ signifies water footprint scarcity indicator: $f(x_{i})<0$ indicates that the water supply of province *i* exceeds its water demand, $f(x_{i})>0$ means that the water supply is below the demand in province *i*, and $f(x_{i})=0$ corresponds to the balanced scenario in province *i*. $\omega_{ti}$ is the typical influencing factors (when t = p, population factor is expressed, when t = a, land factor is expressed, when t = e, economy factor is expressed) of the province *i*, and *α_i_* is a percentage of province *i*’s value of the typical influencing factor in the total value under consideration.

In model (3), the objective function is to iteratively minimize the maximum shortage of adjusted water footprints by a particular influencing factors (population, land, or economy) in the *n* provinces in a lexicographic order. The first equality describes how the water footprint in each province is calculated from different categories (e.g., food cultivation, livestock, industrial production, inter-provincial trade, domestic consumption, and ecology). The second equality depicts the status of water footprint scarcity indicator in each province. The third equality defines the influencing factor ($\alpha_{i}$) as the land area or population or economic output (GDP) in each province ($\omega_{ti}$) relative to the whole area under consideration. The next inequality constraint ensures that the total allocated water footprint adjusted by three influencing factors in each province is within its available water resources, and the last inequality constraint specifies the upper (${\bar{Q}}_{i}$) and lower bounds (${\underline{Q}}_{i}$) for the allocated water footprint for each province. Given the actual water resources distribution in the YREB, $\sum_{i\in N}{\underline{Q}}_{i}\leq \sum_{i\in N}Q_{i}$ is assumed to guarantee the existence of an optimal solution (i.e., the basic needs of all provinces can be satisfied by properly allocating water resources in the whole area). In addition, there exists at least one province *i* such that its original water footprint *x_i_* satisfies $\alpha_{i}\cdot x_{i}\geq Q_{i}$ (i.e., at least one province is in shortage of water resources from a water footprint perspective). This ensures that the optimal solution $Lex\min^{*}$ > 0.

To solve model (1), the lexicographic minimax algorithm is employed to ensure that the allocation conforms to the principle of fairness [30]. The weight factor $\alpha_{i}$ helps to achieve fair, efficient, and sustainable water footprint allocation in the YREB given available water resources [31]. For detailed descriptions of the solution procedure, readers are referred to Appendix A.

#### 3.3.2. Comprehensive Discriminant Index Family

The variation of the LAWF allocation results reflects allocation fairness. To assess fairness of different LAWF allocation schemes under diverse influencing factors, this paper proposes a comprehensive discriminant index family based on the Gini coefficient and the Theil entropy index. This index family aims to analyze the effect of different influencing factors from different angles and helps to interpret the variation of the data.

(1) Gini Coefficient Model

It is assumed that there are *m* groups (or regions) in area *A*, which includes *n* provinces. The mean water footprint across the area is denoted by *μ*. Let *y_i_*, *y_j_* denote the total water footprint of province *i* and *j* and let *N_k_* denote the *k*^th^ region of *n_k_* provinces according to spatial agglomeration (e.g., upstream, midstream, and downstream in the YREB). Then, the ratio of the number of provinces in the *k*^th^ region to the total number in the area is expressed as $r_{k}=n_{k}/n$. Denote the mean water footprint of the *k*^th^ region by *μ_k_*. Then, the ratio of the mean water footprint in the *k*^th^ region relative to the whole area is given by $\lambda_{k}=\mu_{k}/\mu$, and $\lambda_{k}$, $\lambda_{h}$ represent the proportions of region *k* and *h*, respectively. Given that we are considering both the intra- and inter-regional variations, this paper chooses the model proposed by Mookherjee and Shorrocks [32], and calculate the Gini coefficient as follows:

(4) $$\begin{array}{cc} G & =\frac{1}{2n^{2}\mu }\sum_{i}\sum_{j}\left|y_{i}-y_{j}\right|\\ & =\frac{1}{2n^{2}\mu }\sum_{k}\left(\sum_{i\in N_{k}}\sum_{j\in N_{k}}\left|y_{i}-y_{j}\right|+\sum_{i\in N_{k}}\sum_{j\notin N_{k}}\left|y_{i}-y_{j}\right|\right)\\ & =\sum_{k}{\left(\frac{n_{k}}{n}\right)}^{2}\frac{\mu_{k}}{\mu }G_{k}+\frac{1}{2n^{2}\mu }\sum_{k}\sum_{i\in N_{k}}\sum_{j\notin N_{k}}\left|y_{i}-y_{j}\right|, \end{array}$$

where *G_k_* is the Gini value for the *k^th^* group alone. If the range of water footprints in any group *k* does not overlap with that of any other group *h*, then:

(5) $$\sum_{i\in N_{k}}\sum_{j\in N_{k}}\left|y_{i}-y_{j}\right|=n_{k}n_{h}\left|\mu_{k}-\mu_{h}\right|\;h\neq k,$$

and Equation (4) becomes:

(6) $$G=\sum_{k}r_{k}{}^{2}\lambda_{k}G_{k}+\frac{1}{2}\sum_{k}\sum_{h}r_{k}r_{h}\left|\lambda_{k}-\lambda_{h}\right|,$$

Furthermore, when the subgroup water footprints ranges overlap (as will normally be the case), Equation (6) is modified to:

(7) $$G=\sum_{k}r_{k}{}^{2}\lambda_{k}G_{k}+\frac{1}{2}\sum_{k}\sum_{h}r_{k}r_{h}\left|\lambda_{k}-\lambda_{h}\right|+R$$

Equation (7) decomposes the Gini coefficient into three components, where the first term represents the intra-regional difference (hereafter, referred to as *G*_1_), the second term stands for the inter-regional difference (hereafter, referred to as *G*_2_), and the last term gives the residual difference (hereafter, referred to as *R*), respectively. Here the residual term *R* mainly reflects the influence of individual rankings within each region on the Gini coefficient and gauges the degree of interaction among different subgroups. The smaller the R, the smaller the degree of interaction. At the same time, Chen [17] noted that the larger the Gini coefficient *G*, the greater the degree of data variation.

(2) Theil Entropy Model

The Theil entropy can effectively measure the contribution of the intra-regional and inter-regional variations with unbalanced characteristics. In essence, the Theil entropy is a special case of the general entropy [32] and can be expressed as:

(8) $$\left\{ \begin{array}{l} T=T_{1}+T_{2}=\sum_{k}\frac{n_{k}}{n^{2}}\sum_{j}\ln\frac{\mu_{k}}{y_{kj}}+\sum_{k}\frac{n_{k}}{n}\ln\frac{\mu }{\mu_{k}}\\ L=L_{1}+L_{2}=\sum_{k}\frac{n_{k}}{n^{2}}\cdot \frac{\mu_{k}}{\mu }\sum_{j}\frac{y_{kj}}{\mu }\ln\frac{y_{kj}}{\mu }+\sum_{k}\frac{n_{k}}{n}\cdot \frac{\mu_{k}}{\mu }\ln\frac{\mu_{k}}{\mu } \end{array}\right.$$

where *y_kj_* denotes the total amount of the water footprint in the *j*^th^ province in the *k*^th^ region. It has been noted that *T* is more sensitive to changes in the higher end of the data set and *L* is more sensitive to changes in the low end of the data. *T_1_* and *L_1_* represent the intra-regional variations, and *T*_2_ and *L*_2_ represent the inter-regional variations.

(3) Comprehensive Discriminant Index Family

The Gini coefficient is more sensitive to the change of data near the median, while the Theil T index is more sensitive to the change of data in the upper quartile and the Theil L index is more sensitive to the change of data in the lower quartile. Combining Gini and Theil into a comprehensive index family can effectively reduce the impact of information deficiency of traditional discriminant methods and improve the interpretation capability of discriminant results. Therefore, based on the Formulas (5) and (6), this paper employs a comprehensive discriminant index family to analyze the effect of different influencing factors on the LAWF allocation schemes as follows:

(9) $$\left\{ \begin{array}{l} T=T_{1}+T_{2}=\sum_{k}\frac{n_{k}}{n^{2}}\sum_{j}\ln\frac{\mu_{k}}{y_{kj}}+\sum_{k}\frac{n_{k}}{n}\ln\frac{\mu }{\mu_{k}}\\ G=\sum_{k}r_{k}{}^{2}\lambda_{k}G_{k}+\frac{1}{2}\sum_{k}\sum_{h}r_{k}r_{h}\left|\lambda_{k}-\lambda_{h}\right|+R\\ L=L_{1}+L_{2}=\sum_{k}\frac{n_{k}}{n^{2}}\cdot \frac{\mu_{k}}{\mu }\sum_{j}\frac{y_{kj}}{\mu }\ln\frac{y_{kj}}{\mu }+\sum_{k}\frac{n_{k}}{n}\cdot \frac{\mu_{k}}{\mu }\ln\frac{\mu_{k}}{\mu } \end{array}\right.$$

In the formula, the Theil *T* index mainly analyzes data variations near the upper quartile, and its geometric meaning is the conditional information entropy index. *G* aims to gauge data variations near the median and its geometric meaning is the mean difference index. *L* mainly measures the change of data near the lower quartile and its geometric meaning is the logarithmic discrete mean.

(4) Discriminant Rules

Traditional studies [33] argue that 0.3–0.4 is a reasonable interval for the Gini coefficient, but a reasonable interval discriminant criterion has not yet been established for the Theil entropy. On the one hand, the traditional Gini coefficient has difficulty in explaining the rationality of resource allocation variations. On the other hand, there is an essential difference between the influence of the traditional income distribution and the different influencing factors of the LAWF allocations. As such, it is difficult to apply directly the traditional Gini coefficient to assess LAWF allocation schemes. Given water resources’ quasi-public property attribute, equal allocation apparently does not solve the conflict. It is critical to establish a coordinated relationship between ecological protection and economic development. Therefore, the essence of valid discriminant of a LAWF scheme is a holistic gauge of both equalization and differentiation of resource allocation. Firstly, it is trivial to verify that the proposed comprehensive index family in Equation (7) falls within (0,1), i.e., 0≤T≤1, 0≤G≤1, 0≤L≤1. Secondly, when we assess LAWF allocation schemes based on different influencing factors, land, population, and economy, it is necessary to tell which allocation scheme is fairer. To this end, Definition 1 is introduced.

**Definition** **1.** *For two LAWF allocation schemes, s and t, if $0\leq T^{s}\leq T^{t}\leq 1$, then scheme s is more equalitarian than scheme t when the index is sensitive to the provinces with high water footprint. If $0\leq G^{s}\leq G^{t}\leq 1$, then scheme s is more equalitarian than scheme t when the index is sensitive to the provinces with medium water footprint. If $0\leq L^{s}\leq L^{t}\leq 1$, then scheme s is more equalitarian than scheme t when the index is sensitive to the provinces with low water footprint.*

Thirdly, it has been well documented that complete equality is neither possible nor desirable. From a resource allocation perspective, every province has different resource endowments, populations, and economies, leading to different demand for scarce water resources. As such, it is necessary to set a lower bound for the Gini coefficient and Theil indices so that an allocation scheme remains fair and effective.

**Definition** **2.** *For a sufficiently small ε and an allocation scheme s, if T^s^, G^s^, L^s^ satisfy $\{s|0<T^{s}\leq \varepsilon ,0<G^{s}\leq \varepsilon ,0<L^{s}\leq \varepsilon \}$, the actual configuration effect of scheme s is deemed poor as it tends to be an equalitarian but ineffective allocation. For another scheme t, if T^t^, G^t^, L^t^ satisfy $\{t|T^{t}>\varepsilon ,G^{t}>\varepsilon ,L^{t}>\varepsilon \}$, then the actual configuration effect of scheme t is better than that of scheme s as t tends to be fairer and more effective than s for the high, medium, low water load provinces.*

## 4. Results

To explore the different effect of the three influencing factors of land, population, and economy on the LAWF result in the YREB, this paper makes a thorough analysis as detailed below.

### 4.1. Effect Analysis of Heterogeneous Influencing Factors on LAWF

To assess the effect of population, economic output (GDP), and land area on the LAWF, model (1) is employed to obtain the optimized water footprints in the 11 YREB provinces based on the 2013 data. Based on model (1) and the lexicographic minimax algorithm in Appendix A, we obtain the result as shown in Table 2. In Table 2, ***WF_o_*** denotes original water footprints, ***WF_p_*** denotes the optimized water footprint allocation schemes with population as the influencing factor, ***WF_e_*** denotes the optimized water footprint allocation schemes with economy as the influencing factor, and ***WF_a_*** denotes the optimized water footprint allocation schemes with land area as the influencing factor. 

**Figure 2:**
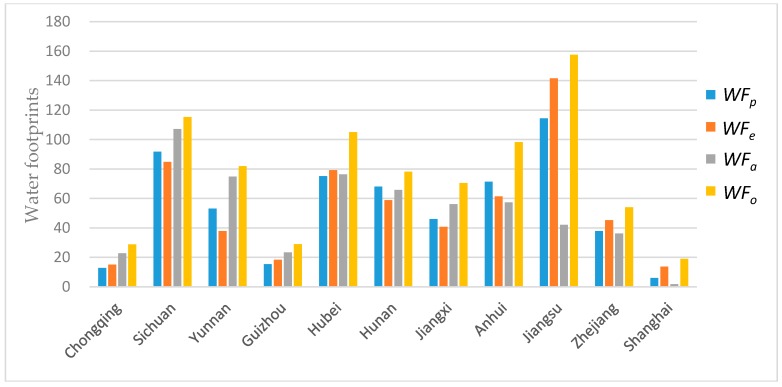
Effect of Heterogeneous Influencing Factors on LAWF.

As shown in Table 2 and Figure 2, compared with the original *WF_o_*, the average reductions of water footprints under population (*WF_p_*), economy (*WF_e_*), and land area (*WF_a_*) are 29.33%, 28.75%, 32.67%, respectively. In the allocation scheme *WF_p_*, the relationship between the mean of optimization results of the upstream, midstream, and downstream in the YREB is $\bar{WF_{m}}>\bar{WF_{d}}>\bar{WF_{u}}$. In the allocation scheme *WF_e_*, the relationship between the mean of optimization results of the upstream, midstream, and downstream in the YREB is $\bar{WF_{d}}>\bar{WF_{m}}>\bar{WF_{u}}$. In the allocation scheme *WF_a_*, he relationship between the mean of optimization results of the upstream, midstream, and downstream in the YREB is $\bar{WF_{m}}>\bar{WF_{u}}>\bar{WF_{d}}$.

For the upstream in the YREB, the relationship between the mean of optimization results of three schemes is $WF_{e}<WF_{p}<WF_{a}<WF_{o}$. For the midstream in the YREB, the relationship between the mean of optimization results of three schemes is $WF_{e}<WF_{p}<WF_{a}<WF_{o}$. For the downstream in the YREB, the relationship between the mean of optimization results of three schemes is $WF_{a}<WF_{p}<WF_{e}<WF_{o}$.

Overall, the optimal allocation schemes, *WF_p_*, *WF_e_*, and *WF_a_*, are affected by both the original water footprint and the corresponding influencing factor. On the one hand, all three schemes effectively reduce the water footprints in the YERB. On the other hand, in the allocation scheme *WF_a_* and *WF_p_*, the mean of water footprint quota in the midstream is the largest. In the allocation scheme *WF_e_*, the mean of water footprint quota in the downstream is the largest. The upstream and midstream with the *WF_a_* scheme will get the greatest water footprint quotas. The downstream with the *WF_e_* scheme will get the greatest water footprint quotas.

### 4.2. Results of Comprehensive Discriminant Index Family

**Figure 3:**
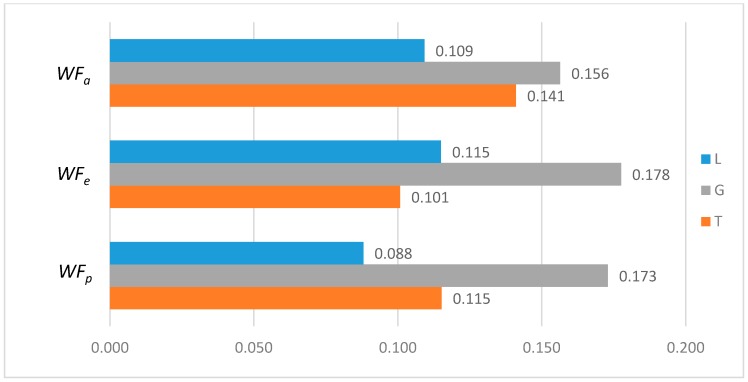
Results of Comprehensive Discriminant Index Family.

To further analyze the differences between the LAWF optimal schemes under the three influencing factors across the upstream, midstream, and downstream in the YERB, we apply the proposed comprehensive discriminant index family to assess their impact on the allocation fairness. The results are shown in Figure 3.

Denoting the *T* index of *WF_a_*, *WF_p_*, *WF_e_* by *T*(A), *T* (P), and *T* (E), respectively, we can see from Figure 3 that T(E)<T(P)<T(A). According to *Definition 1 and 2*, for the provinces with high water footprint quotas (top three provinces ranked by the optimized water footprint quotas), the *WF_e_* optimization scheme has the smallest difference in water footprints among provinces, while the *WF_a_* optimization scheme has more significant differences.

Denoting the *L* index of *WF_a_*, *WF_p_*, and *WF_e_* by *L* (A), *L* (P), and *L* (E), respectively, from Figure 3, we have L(P)<L(A)<L(E). According to *Definition 1 and 2*, for the provinces with low water footprint quotas (last three provinces ranked by the optimized water footprint quotas), the *WF_p_* optimization scheme has the smallest difference in water footprints among provinces, while the *WF_e_* optimization scheme has the strongest discreteness.

Denoting the *G* index of *WF_a_*, *WF_p_*, *WF_e_*, respectively, we know from Figure 3 that G(A)<G(P)<G(E). According to *Definition 1 and 2*, for the provinces with medium water footprint quotas (fourth to eighth provinces ranked by the optimized water footprint quotas), the *WF_a_* optimization scheme has the smallest difference in water footprints among provinces, while the *WF_e_* optimization scheme has the strongest discreteness.

In summary, by comparing the *T*, *L*, and *G* indices of the three allocation schemes, *WF_p_*, *WF_e_*, and *WF_a_*, some conclusions can be drawn. If the key fairness concern is to reduce the difference of water footprint allocations among the 11 YREB provinces, the *WF_e_* optimal scheme arises as the best choice for provinces with high water footprints, the *WF_a_* optimal scheme can better meet the needs of provinces with medium water footprints, while the *WF_p_* optimal scheme works the best for provinces with low water footprints. The heterogeneous demand of different water-load provinces for equitable allocation makes it impossible to achieve a global optimal solution under any single factor. As such, it is necessary to construct a water footprint allocation model considering multiple factors, population, economy and land area.

## 5. Discussion

### 5.1. Analysis of Heterogeneous Influencing Factors on LAWF

The upstream of the YREB consists of the provinces of Sichuan, Guizhou, Yunnan and the municipality of Chongqing. Among the three optimized water footprints of *WF_p_*, *WF_e_* and *WF_a_*, the upstream four provinces/municipality have the largest water footprints according to the *WF_a_* optimization scheme, compared with the other two schemes. As shown in Table 1, the total land area of the upstream region accounts for 55.09% of the YREB, with 32.74% of the total population, and 22.60% of the GDP. Therefore, the *WF_a_* optimization scheme provide the greatest water footprint quota to the upstream region.

The midstream region includes the provinces of Hubei, Hunan, and Jiangxi. On the one hand, the order of the original water footprint of the three provinces in the middle reaches is Hubei’s > Hunan’s > Jiangxi’s. The ranking in three optimized water footprints of *WF_p_*, *WF_e_* and *WF_a_* is also Hubei’s > Hunan’s > Jiangxi’s. On the other hand, Hubei can obtain the greatest water footprint quota according to *WF_e_* optimization scheme, compared with the *WF_p_* and *WF_a_* schemes. Hunan has the highest water footprint available according to the *WF_p_* optimization scheme. Jiangxi possesses the largest water footprint quota with *WF_a_* optimization scheme. This indicates that for the midstream region of YERB, the three optimization schemes of *WF_p_*, *WF_a_* and *WF_e_* are highly dependent on the relative proportion of population, economy and land area of the provinces in the midstream region. As shown in Table 1, the total land area in the midstream region accounts for 27.71% of the YREB, with 29.69% of the total population, and 24.47% of the GDP. The consistent proportion of the three major impact factors is the main reason for the similar results of the LAWF in the midstream region.

The downstream region of the YREB mainly comprises three provinces, Jiangsu, Zhejiang, Anhui, and Shanghai municipality. As shown in Table 2, among the three optimized water footprints of *WF_p_*, *WF_e_* and *WF_a_*, Jiangsu, Zhejiang, and Shanghai all obtain the greatest water footprint quota according to the *WF_e_* scheme. But Anhui obtain the highest water footprint quota according to the *WF_p_* scheme. From Table 1, one can determine that the proportions of population, GDP and land area in Anhui Province are 10.37%, 7.34%, 6.85%, respectively. The proportion of Anhui’s population factor in YERB is the highest among the three influencing factors. Therefore, for Anhui Province, more water footprint can be obtained according to the *WF_p_* optimization scheme, while economic factors account for the highest proportion in the other three provinces. The total land area of the downstream region accounted for 17.20% of the YREB, with 37.57% of the total population, and 52.93% of the GDP. Therefore, in general, the downstream of YERB can obtain the highest water footprint quota according to the *WF_e_* optimization scheme.

### 5.2. Analysis of the Intra-Regional and Inter-Regional Variations of T, L, G index of LAWF

In order to analyze the differences among regions, the inter-group and intra-group variations of the *T*, *L*, *G* index, and their corresponding proportions are calculated. The result is shown in Table 3 below.

By comparing and analyzing the contribution degree of the *T*, *L* and G indices in the intra-region and inter-region, it can help to clarify the impact mechanism of the three major factors of population, economy and land on the allocation of water footprints within and between the upper and lower regions. The contribution degree of the intra-regional variation of the *T* index (denoted by $D_{T_{1}}$) is determined by $T_{1}/T\times 100\%$. Similarly, the contribution degree of the intra-regional variation of the *L* index (denoted by $D_{L_{1}}$) is calculated by $L_{1}/L\times 100\%$, and the contribution degree of the intra-regional variation of the *G* index (denoted by $D_{G_{1}}$) by $G_{1}/G\times 100\%$. Meanwhile, the contribution degree of the inter-regional variation of the *T* index (denoted by $D_{T_{2}}$) is determined by $T_{2}/T\times 100\%$, the contribution degree of the inter-regional variation of the *L* index (denoted by $D_{L_{2}}$) by $L_{2}/L\times 100\%$, and the contribution degree of the inter-regional variation of the *G* index (denoted by $D_{G_{2}}$) by $G_{2}/G\times 100\%$. One can denote the $D_{T_{1}}$ of *WF_a_*, *WF_p_*, *WF_e_* by $D_{T_{1}}\left(A\right)$, $D_{T_{1}}\left(P\right)$ and $D_{T_{1}}\left(E\right)$; the $D_{L_{1}}$ of *WF_a_*, *WF_p_*, *WF_e_* by $D_{L_{1}}\left(A\right)$, $D_{L_{1}}\left(P\right)$ and $D_{L_{1}}\left(E\right)$; and the $D_{G_{1}}$ of *WF_a_*, *WF_p_*, *WF_e_* by $D_{G_{1}}\left(A\right)$, $D_{G_{1}}\left(P\right)$ and $D_{G_{1}}\left(E\right)$, respectively. From Table 3, one can see that $D_{T_{1}}\left(A\right)<D_{T_{1}}\left(E\right)<D_{T_{1}}\left(P\right)$, $D_{L_{1}}\left(A\right)<D_{L_{1}}\left(E\right)<D_{L_{1}}\left(P\right)$ and $D_{G_{1}}\left(A\right)<D_{G_{1}}\left(E\right)<D_{G_{1}}\left(P\right)$. Denoting the $D_{T_{2}}$ of *WF_a_*, *WF_p_*, *WF_e_* by $D_{T_{2}}\left(A\right)$, $D_{T_{2}}\left(P\right)$ and $D_{T_{2}}\left(E\right)$, the $D_{L_{2}}$ of *WF_a_*, *WF_p_*, *WF_e_* by $D_{L_{2}}\left(A\right)$, $D_{L_{2}}\left(P\right)$ and $D_{L_{2}}\left(E\right)$, the $D_{G_{2}}$ of *WF_a_*, *WF_p_*, *WF_e_* by $D_{G_{2}}\left(A\right)$, $D_{G_{2}}\left(P\right)$ and $D_{G_{2}}\left(E\right)$, respectively. It is shown in Table 3 that $D_{T_{2}}\left(P\right)<D_{T_{2}}\left(E\right)<D_{T_{2}}\left(A\right)$, $D_{L_{2}}\left(P\right)<D_{L_{2}}\left(E\right)<D_{L_{2}}\left(A\right)$ and $D_{G_{2}}\left(P\right)<D_{G_{2}}\left(E\right)<D_{G_{2}}\left(A\right)$. This indicates that the intra-regional variation of *WF_p_* optimization scheme has a greater contribution to the total variation in the YERB. The inter-regional variation of the *WF_a_* optimization scheme has a greater contribution to the total variation.

It should be pointed out that the residual index of the *G* index can be used to characterize the overlap degree of the inter-regional water footprint [34], as an important indicator for measuring the fairness of the configuration. Denoting the *R* of *WF_a_*, *WF_p_*, *WF_e_* by *R(A)*, *R(P)*, *R(E)*, respectively. The results in Table 3 indicates that R(A)<R(E)<R(P). It can be seen that the inter-regional water footprint under the *WF_p_* optimization scheme has the highest overlap, which is mainly due to the fact that the water footprints of Sichuan, Hubei, and Jiangsu with *WF_p_* optimization scheme are significantly larger than those of other provinces in upstream, midstream, and downstream, respectively. Therefore, the overlap of the inter-regional water footprint under the *WF_p_* optimization scheme is relatively high. In other words, the difference in inter-regions under the *WF_p_* scheme is the smallest, which is consistent with the sorting results of *L*_2_, *T*_2_, and *G*_2_. Conversely, the difference in inter-regions under the *WF_a_* scheme is the largest. In summary, the three optimization schemes of *WF_p_*, *WF_e_* and *WF_a_* differ greatly in the contribution of intra-regional and inter-regional variations. The contribution of intra-regional variation in *WF_p_* optimization scheme is the largest of the three schemes. The contribution of inter-regional variation in *WF_a_* optimization scheme is the largest of the three schemes. In the *WF_p_* optimization scheme, the difference in the inter-regions is the smallest, and when the *WF_a_* optimization scheme is used, the difference in the inter-regions is the largest.

### 5.3. LAWF under Multi-Factor Coupling

Considering the difference in water footprint allocation preferences of different regions and the complex mechanism of population, economic and land area factors for water footprint allocation, this paper constructs the LAWF model based on the optimization method for integrating multiple factors (hereafter, referred to as *WF_OM_*) and the LAWF model based on the entropy method for integrating multiple factors (hereafter, referred to as *WF_EM_*). Among them, the *WF_OM_* is utilized to select the highest proportion of each province in YREB among the three factors of population, economy and land area. That is, $\omega_{oi}=Max(p_{i}/\sum p_{i},e_{i}/\sum e_{i},a_{i}/\sum a_{i})$, where *p_i_* represents the population of province *i*, *e_i_* means the GDP of province *i*, *a_i_* implies the land area of province *i*. *ω_oi_* stands for multiple factors of province *i* based on the optimization method. The *WF_EM_* refers to the calculation of the information entropy [35] of the respective population, economy and land area of 11 provinces as the weight coefficient of the three factors. According to Table 1, after standardizing the original data, the weight coefficient of the three factors are calculated as follows: population: GDP: land area = 0.13: 0.33: 0.54. That is, in Equation (1), *ω_ei_* = 0.13*p_i_* + 0.33*e_e_* + 0.54*a_i_*. *ω_ei_* represents multiple factor of province *i* based on the entropy method. In order to characterize the water supply balance in the region, we introduce the supply-demand balance index of water resources carrying capacity (hereafter, referred to as ***IWSD***) [36]. The *IWSD* is determined by $\frac{WA-WF_{\lambda }}{WA}$, where *WA* is total available water resources of each province. When *λ* = o, it represents the original *IWSD*. When *λ* = *OM*, it stands for the *IWSD* after *WF_OM_* optimization scheme. When *λ* = *EM*, it is the *IWSD* after *WF_EM_* optimization scheme. Obviously, when the available water resources is less than the water footprint of the social and economic system, *IWSD* < 0. This means that the water resources available in this area do not have the ability to support the social and economic system of this scale. Conversely, when the available water resources is greater than the water footprint of the social and economic system, *IWSD* ≥ 0. This indicates that the water resources available in the area have the ability to support such a scale of socio-economic system, and the supply and demand are in good condition. The results of the LAWF under multi-factor coupling are shown in Table 4 below. In Table 4, ***IWSD_O_*** denotes the original *IWSD. **IWSD******_OM_*** denotes the *IWSD* after *WF_OM_* optimization scheme. ***IWSD******_EM_*** denotes the *IWSD* after *WF_EM_* optimization scheme.

It can be seen from Table 4 that compared with the original situation, the *WF_OM_* and *WF_EM_* schemes constructed in this paper can significantly reduce the total water footprint allocation of the YREB, and the total water footprint allocation is reduced by 26.2% and 23.3% respectively. The *IWSD* of each province is effectively improved, the total *IWSD* of YREB increased from 0.24 to 0.44 or 0.42 respectively. The number of provinces with negative *IWSD* have been reduced, which further alleviates the overburden water use in the YREB. If the goal of saving water resources is to be achieved, the *WF_OM_* scheme has a better effect. The *WF_EM_* scheme has a better effect if operability is targeted.

**Figure 4:**
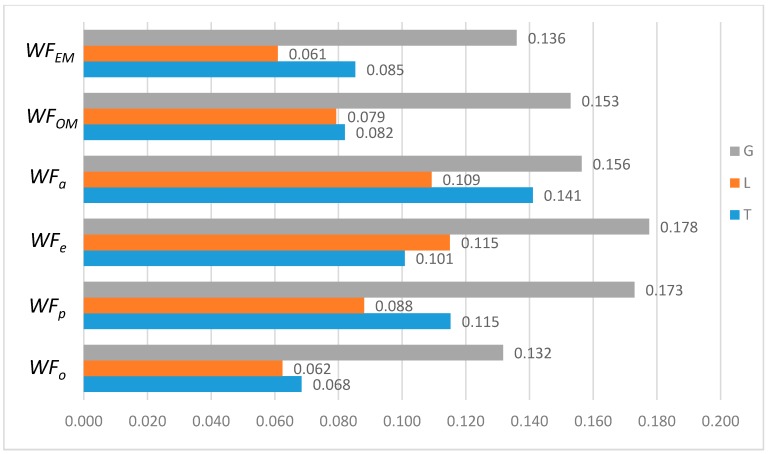
*T*, *L*, *G* index of the LAWF scheme under multi-factor coupling.

In order to further compare the differences between *WF_OM_*, *WF_EM_* and among *WF_p_*, *WF_e_*, and *WF_a_*, the *T*, *L*, and *G* indices of *WF_OM_* and *WF_EM_* are calculated, as shown in Figure 4 below.

As shown in Figure 4, the *T* index of *WF_OM_* is smaller than the *T* index of *WF_EM_*, while the *L* and *G* index of *WF_EM_* is smaller than those of *WF_OM_*. This indicates that *WF_EM_* has greater differences for provinces with high water footprint quotas, and *WF_OM_* has greater differences for provinces with low and medium water footprint quotas. This is for the two schemes have different perspectives of concern. The *WF_OM_* mainly focuses on the factors of the optimum proportion of 11 provinces, and obtains the comprehensive factor matrix by selecting the factors of the optimum proportion of each province. *WF_EM_* is based on the relative discreteness of three influencing factors in 11 provinces, and forms the weight coefficients of multi-factor coupling effect.

In comparison to the single factor optimization schemes, one can see that the *T*, *L*, and *G* indices of *WF_OM_* and *WF_EM_* schemes are significantly lower than those of *WF_p_*, *WF_e_*, and *WF_a_* schemes. It shows that the optimized water footprint of each province by using the LAWF under multi-factor coupling is more equalitarian. Compared with the original situation, the *WF_OM_* and *WF_EM_* schemes has larger configuration difference. But according to *Definition 2*, the original situation is deemed poor as it tends to be an equalitarian but ineffective allocation. It is by differential configuration that *WF_OM_* and *WF_EM_* reduce the total water footprint allocation, improve the supply-demand balance index of water resources carrying capacity, and have practical significance for solving the imbalance between supply and demand of water resources allocation in YREB.

## 6. Conclusions

In summary, in view of the water footprint lexicographic optimization, in this paper, land area, population and GDP are selected as main influence factors of LAWF. The Gini coefficient and the Theil entropy index are utilized to construct the comprehensive discriminant index family and discriminant rules. Taking the section data of 11 provinces in the YREB in 2013 as an example, the discriminant analysis of the impact of the three influencing factors on the LAWF was performed. The results shows that: (1) Using the comprehensive discriminant index family to analyze *WF_p_*, *WF_e_*, and *WF_a_* scheme, it can be seen that for the provinces with high water footprint quotas, the *WF_e_* scheme is more equalitarian. For the provinces with low water footprint quotas, the *WF_a_* scheme is more equalitarian. For the provinces with medium water footprint quotas, the *WF_p_* scheme is more equalitarian. (2) The contribution of intra-regional variation in the population-based LAWF scheme is the largest of the three schemes. The inter-regional variation contributed the largest in the land area-based LAWF scheme. (3) The two schemes under multi-factor coupling is more equalitarian than the three single-factor schemes. Compared with the original situation which is an equalitarian but ineffective allocation, the *WF_OM_* and *WF_EM_* schemes have greater effect on the improvement of the supply-demand balance of water resources carrying capacity. The contributions are twofold. First, this paper establishes a comprehensive discriminant index family and discriminant rules based on Gini coefficient and the Theil entropy to evaluate the lexicographic allocation scheme under different influence factors, and provides some theoretical support for the government’s water resources allocation work. Second, this research reveals that in the process of water resources allocation, comprehensive evaluation and analysis should be carried out according to regional population structure, resource endowment and industrial structure. The defect of the population, economy and land area factors acting alone should be resolved by designing a weighting system, in order to optimize the allocation of water resources. It can improve the utilization efficiency of water resource, and protect the water security, economic security, and social security measures of the YREB.

This study can be further extended along several different directions. First, grey water footprints are not accounted because of the unavailability of reliable data. Second, the FAO crop coefficient data are used to determine agricultural water footprints since region-specific data are difficult to obtain for local crops in the YREB. These issues warrant further research.

## Figures and Tables

**Table 1:** The original data of the YREB.

Province	Land Area (km^2^)	Population (10,000)	GDP (million RMB)	Available Water Resources (billion m^3^)	Total Water Consumption (billion m^3^)
Chongqing	82,300	2970.00	12,656.69	47.43	8.39
Sichuan	481,400	8107.00	26,260.77	247.03	24.25
Yunnan	383,300	4686.60	11,720.91	170.67	14.97
Guizhou	176,000	3502.22	8006.79	75.94	9.20
Hubei	185,900	5799.00	24,668.49	79.01	29.18
Hunan	211,800	6690.60	24,501.67	158.20	33.25
Jiangxi	167,000	4522.20	14,338.50	142.40	26.48
Anhui	139,700	6029.80	19,038.90	58.56	29.60
Jiangsu	102,600	7939.49	59,161.75	28.35	57.67
Zhejiang	102,000	5498.00	37,568.49	93.13	19.83
Shanghai	6300	2415.15	21,602.12	2.80	12.32
Upstream mean	280,750	4816.40	14,661.29	135.27	14.20
Midstream mean	188,233	5670.60	21,169.55	126.54	29.64
Downstream mean	87,650	5470.61	34,342.82	45.71	29.86

**Table 2:** The optimized and the original water footprints in the YERB (billion m^3^).

	*WF_o_*	*WF_p_*	*WF_e_*	*WF_a_*
Chongqing	28.83	12.81	15.04	22.78
Sichuan	115.29	91.83	84.77	107.22
Yunnan	82.00	53.13	37.84	74.79
Guizhou	29.00	15.34	18.42	23.45
Hubei	105.10	75.19	79.31	76.41
Hunan	78.21	68.10	58.89	65.76
Jiangxi	70.41	46.03	40.69	56.20
Anhui	98.25	71.37	61.46	57.42
Jiangsu	157.59	114.36	141.47	42.17
Zhejiang	54.02	37.81	45.32	36.16
Shanghai	19.04	6.03	13.71	1.74
Sum of YREB	837.951	592.01	596.92	564.09
Upstream mean	63.78	43.28	39.02	57.06
Midstream mean	84.57	63.11	59.63	66.12
Downstream mean	82.23	57.39	65.49	34.37
Decreasing mean of YREB	-	29.33%	28.75%	32.67%

**Table 3:** The intra-regional and inter-regional variations of *T*, *L*, *G* index of LAWF.

	*T* index	*L* index	*G* index
***WF_p_***	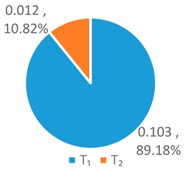	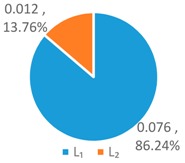	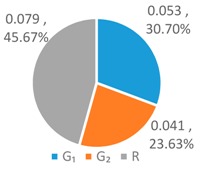
***WF_e_***	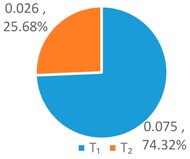	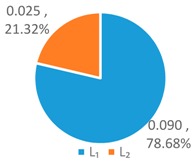	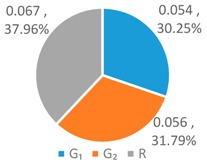
***WF_a_***	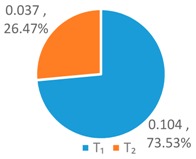	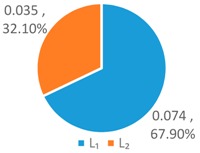	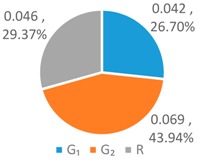

**Table 4:** The results of the LAWF under multi-factor coupling.

Province	*WA* (billion m^3^)	*WF_O_* (billion m^3^)	*WF_OM_* (billion m^3^)	*WF_EM_* (billion m^3^)	*IWSD_O_*	*IWSD_OM_*	*IWSD_EM_*
Chongqing	47.43	28.83	22.29	28.10	0.39	0.53	0.41
Sichuan	247.03	115.29	103.07	112.39	0.53	0.58	0.55
Yunnan	170.67	82.00	67.66	79.30	0.52	0.60	0.54
Guizhou	75.94	29.00	17.96	19.08	0.62	0.76	0.75
Hubei	79.01	105.10	58.91	81.41	−0.33	0.25	−0.03
Hunan	158.20	78.21	55.86	63.69	0.51	0.65	0.60
Jiangxi	142.40	70.41	42.16	50.84	0.51	0.70	0.64
Anhui	58.56	98.25	61.25	61.06	−0.68	−0.05	−0.04
Jiangsu	28.35	157.59	134.68	97.70	−4.56	−3.75	−2.45
Zhejiang	93.13	54.02	41.75	42.01	0.42	0.55	0.55
Shanghai	2.80	19.04	11.52	6.94	−5.80	−3.11	−1.48
Sum	1103.52	837.75	617.11	642.52	0.24	0.44	0.42
Decreasing mean of YREB	-	-	26.2%	23.3%	-	-	-

## Appendix A: Lexicographic Algorithm

To solve model (1), the lexicographic algorithm in Liu et al. [37] is employed. To make the paper self-contained, the algorithm therein is replicated below. To describe the lexicographic minimax solution process, the following two definitions are needed:

**Definition** **A1** [38]. *For two vectors α = (α_1_, α_2_, …, α_m_) and β = (β_1_, β_2_, …, β_m_), if there exists a positive integer k, 1 ≤ k ≤ m, such that α_i_ = β_i_ for any 1 ≤ i ≤ k, and α_k_ < β_k_, then vector α is strictly less than β in a lexicographic order denoted by α < _L_ β. Similarly, a weak lexicographic order α ≤ _L_ β is defined as α < _L_ β or α = β.*

**Definition** **A2** [38]. *If a non-increasing lexicographic solution vector $x=(x_{1},x_{2},\ldots ,x_{n})$ is less than or equal to any other feasible configuration solution, then the feasible configuration solution $x\in {\mathbb{R}}_{+}^{n}$ is called the lexicographic minimax solution.*

The key to tackling a Leximinimax problem is to solve the following minimax problem successively by using the lexicographic method:

(A1) $$\begin{array}{l} P*=\min[\underset{i\in I}{\max}(\alpha_{i}\cdot f(x_{i}))]\\ s.t.\;\sum_{i\in I}\alpha_{i}\cdot f(x_{i})\leq Q_{i},\forall i\in I\\ \;L_{i}\leq f(x_{i})\leq U_{i},\forall i\in I \end{array}$$

The algorithm presented next follows the basic idea in References [38,39]. Their original concern is to address resource allocation in producing multiple products. In this paper, fair allocation of provincial water footprints under limited water supply constraints can be characterized as a single resource allocation in a single period with multiple stakeholders. Luss’s algorithm is a handy tool to solve our problem. However, because water footprints are shared resources, the solution process of our lexicographic minimax allocation differs from the traditional algorithm in the following aspects:

(1) *Assumptions*: Due to the difference between production resources (e.g., capital and human resources) and natural resources (e.g., land and water resources), traditional industrial production lexicographic minimax problems typically employ cumulative variables, while this paper uses piecewise continuous variables.
(2) *Decision variables*: In the traditional algorithm, decision variables are production quantities, which consume limited resources in the production process and are suitable for enterprise production planning. On the other hand, decision variables in this paper are water footprints, which are appropriate for allocating provincial water resources under government regulation and market mechanisms.
(3) *Solution procedure*: The original solution procedure mainly uses constraints to internalize multiple resources, and aims at solving the lexicographic minimax problem with multiple subjects and multiple periods. Given that our decision variables are water footprints, the algorithm in this paper is designed for lexicographic minimax problems for a single limited resource with multiple subjects.

This paper enhances the applicability of the traditional algorithm by adapting it to handle equitable allocation of provincial water footprints with a single shared resource. Next, the procedure to solve the proposed lexicographic minimax model can be described as follows:

Step 0: Initialize: $\tilde{L_{i}}=L_{i}\;\mathrm{and}\;\tilde{U_{i}}=U_{i}\;\mathrm{for}\;\mathrm{all}\;i\in I$.

Step 1: Compute:

$S_{i}=\sum_{i\in I}\left(x_{i}-Q_{i}\right),\forall i\in I$

$R_{i}=\sum_{i\in I}(WF_{i}/\alpha_{i}),\forall i\in I$

For province *i*’s available water quantity *Q_i_*, remove all *i*’s such that $S_{i}\leq 0$.

If all *Q_i_* = 0, let $x_{i}=WF_{i},\forall i\in I$, then k=0, go to Step 8.

Step 2: Calculate:

$$T_{i}=\sum_{i}WF_{i}-a,\forall i\in I$$

$$M_{i}=\frac{\omega_{i}}{\sum \omega_{i}}\times \sum WF_{i},\forall i\in I$$

$$avg_{i}=\frac{a}{\sum \omega_{i}},\forall i\in I$$

$$k_{i}=T_{i}/R_{i},\forall i\in I.\;\mathrm{For}\;R_{i}>0,\;\mathrm{let}\;k=\max\{k_{i}\}.$$

Step 3: Determine: $x_{i}^{*}=x_{i}(1-k/\alpha_{i}),\forall i\in I$.

Step 4: If *WF_i_* × (1–*k*/*a_i_*) ≥ MIN(*WF_i_*, *M_i_*), ∀i∈I, let *x_i_* = MIN(*WF_i_*, *M_i_*), ∀i∈I.

Step 5: If MIN(*WF_i_*, *M_i_*) ≤ *avg_i_* × *_ωi_*, ∀i∈I, go to Step 6, else go to Step 7.

Step 6: If *WF_i_* × (1–*k*/*a_i_*) ≤ 0, ∀i∈I, let *x_i_* = MIN(*WF_i_*, *M_i_*), ∀i∈I.

Step 7: If *WF_i_* × (1–*k*/*a_i_*) ≤ *avg_i_* × *_ωi_*, ∀i∈I, let *x_i_* = *avg_i_* × *_ωi_*, ∀i∈I.

Step 8: For the minimax problem in each iteration, perform the following calculation:

   $x_{i}=WF_{i}\;for\;all\;i\in I,\forall Q_{i}=0$;

   $P^{*}=\max[k,\underset{i}{\max}(\alpha_{i}\cdot f(x_{i}))]$.

Step 9: Adjust *Q_i_* according to the minmax problem result during the previous iteration, and remove *i*’s such that *i* ∈ {*i*:*R_i_* = 0}.

Step 10: Update constraints ${\tilde{L}}_{i}$ and ${\tilde{U}}_{i}$,

Remove *i*’s such that $i\in \{i:|{\tilde{L}}_{i}={\tilde{U}}_{i}\}$, update $Q_{i}$.

Remove $i\in \{i:|\sum {\tilde{L}}_{i}=Q_{i}\}$. If all *i*’s have been removed, stop.

Otherwise, define a new *P** problem with remaining i∈I, and go back to Step 0.

## Appendix B: Raw Data for Water Footprint Accounting

The tables below show the data of agricultural and non-agricultural water footprints of the 11 provinces and municipalities in the YREB. Table A1 shows the raw data and final calculation agricultural water footprints including cultivated crops and livestock husbandry water footprints. Table A2 shows the data of non-agricultural water footprints, which consist of industrial, domestic, and urban greening water footprints.

**Table A1:** The data for agricultural water footprints in the YREB (unit: 100 million m^3^).

	Chongqing	Sichuan	Yunnan	Guizhou	Hubei	Hunan	Jiangxi	Anhui	Jiangsu	Zhejiang	Shanghai
Wheat	3.92	48.45	7.59	5.77	52.10	1.39	0.34	187.81	214.76	3.53	0.00
Barley	0.00	0.00	0.00	0.00	0.00	0.00	0.00	0.00	2.42	0.50	0.00
Broad bean	0.00	0.00	0.00	0.00	0.00	0.00	0.00	0.00	1.88	1.24	0.00
Paddy	58.36	181.29	53.46	36.85	231.38	330.44	284.57	70.84	80.73	96.89	0.00
Maize	16.78	45.74	53.53	19.97	19.49	13.14	0.91	34.93	17.75	2.41	0.00
Sorghum	0.14	0.00	0.00	0.00	0.00	0.00	0.00	0.00	0.00	0.00	0.00
Potato	7.70	9.11	7.53	7.64	3.44	5.32	0.00	1.42	1.40	1.78	0.00
Soybean	7.55	10.13	3.87	1.34	3.76	0.00	6.45	24.62	13.17	4.94	29.45
Cotton	0.00	1.37	0.00	0.00	54.34	23.13	15.47	30.23	24.32	3.39	0.49
Peanut	2.38	13.34	2.08	2.00	10.83	5.52	11.25	23.49	9.42	1.63	0.00
Rapeseed	7.38	43.69	12.06	13.74	26.05	29.58	14.36	27.57	26.62	7.44	0.36
Sesame	0.00	6.11	0.00	0.00	15.65	1.78	4.40	8.07	0.04	0.02	0.00
Sugarcane	0.00	5.98	204.32	17.04	3.48	12.01	8.15	0.00	0.83	0.00	0.00
Mint	0.00	0.00	0.00	0.00	0.00	0.00	0.00	0.00	0.00	0.00	0.00
Vegetables	21.24	225.26	42.26	27.01	49.74	49.37	25.65	0.00	256.65	32.04	7.17
Tobacco leaf	0.00	0.95	9.76	0.79	0.79	1.24	0.25	0.21	0.00	0.02	0.00
Melon and fruit	9.66	26.59	38.71	8.72	6.67	9.22	11.39	15.10	89.04	17.20	2.00
Tea leaf	0.00	0.92	46.04	0.54	0.00	0.00	0.00	0.64	0.00	0.00	0.00
**Sum (cultivated crops)**	**135.09**	**618.93**	**481.21**	**141.41**	**477.71**	**482.13**	**383.19**	**424.94**	**739.01**	**173.04**	**39.47**
**Livestock products**											
Pork	40.16	181.14	166.58	59.76	168.11	156.46	88.29	114.80	125.27	78.09	11.12
Beef	10.74	58.32	68.17	21.00	40.66	11.82	26.42	36.56	8.37	10.58	0.75
Lamb	0.00	23.44	13.18	2.96	15.36	0.66	1.50	29.41	17.27	11.48	0.72
Poultry	16.50	82.29	0.00	10.60	60.29	0.00	55.17	96.37	187.47	121.44	13.54
Honey	0.53	0.95	0.11	0.05	0.55	0.00	0.36	0.49	0.13	2.41	0.00
Egg	21.78	72.30	21.81	6.50	146.36	0.00	50.95	124.91	212.87	53.41	6.83
Milk	1.46	19.82	18.15	1.31	6.47	0.00	4.45	41.18	22.04	7.43	50.57
Cocoon	0.38	3.32	0.00	0.00	0.00	0.00	0.00	0.00	0.00	1.94	0.00
**Sum (Livestock products)**	**91.55**	**441.57**	**288.00**	**102.17**	**437.81**	**168.94**	**227.14**	**443.72**	**573.43**	**286.78**	**83.53**
**Sum (Agricultural WF)**	**226.65**	**1060.50**	**769.21**	**243.58**	**915.52**	**651.07**	**610.33**	**868.66**	**1312.44**	**459.83**	**123.00**

**Table A2:** The data for non-agricultural water footprints in the YREB (unit: 100 million m^3^).

	Chongqing	Sichuan	Yunnan	Guizhou	Hubei	Hunan	Jiangxi	Anhui	Jiangsu	Zhejiang	Shanghai
Industrial output value (100 million RMB)	5249.65	11,471.57	3767.58	2686.52	10,531.37	9996.6814	6437.9865	8928	25,612.23	16,368.43	7236.69
Industrial water consumption (100 million m^3^)	36.70	44.70	24.6	27.7	90.20	87.7	61.3	91.20	238	55.70	67.20
Product WF (100 million m^3^)	36.70	44.70	24.6	27.7	90.20	87.7	61.3	92.70	238	55.70	66.20
Import virtual water	42.06	30.5	24.16	26.18	46.12	37.19	49.15	39.18	46.18	40.19	34.19
Export virtual water	37.16	29.46	19.46	24.75	42.18	38.32	46.15	51.63	76.19	64.53	59.15
Trade water footprint (100 million m^3^)	4.90	1.04	4.70	1.43	3.94	−1.13	3.00	−12.45	−30.01	−24.34	−24.96
**Sum (Industrial WF)**	**41.60**	**45.74**	**29.30**	**29.13**	**94.14**	**86.57**	**64.3**	**78.75**	**207.99**	**31.36**	**42.24**
Domestic water consumption	19.10	42.50	19.50	16.60	40.70	41.80	27.40	30.90	52.80	43.80	24.40
Urban greening coverage	0.90	4.20	2.00	0.70	0.60	2.70	2.10	4.20	2.70	5.20	0.80
**Sum (Non-agricultural WF)**	**61.60**	**92.44**	**50.80**	**46.43**	**135.44**	**131.07**	**93.80**	**113.85**	**263.49**	**80.36**	**67.44**

## References

[1] UNESCO (2017). The United Nations World Water Development Report 2017.

[2] Hoestra A.Y. (2003). Virtual Water Trade: Proceedings of the International Expert Meeting on Virtual Water Trade [A].

[3] Allan J.A. (1998). Virtual Water: A Strategic Resource Global Solutions to Regional Deficits. Groundwater.

[4] Feng K., Hubacek K., Minx J., Siu Y.L., Chapagain A., Yu Y., Guan D., Barrett J. (2010). Spatially Explicit Analysis of Water Footprints in the UK. Water.

[5] Hoekstra A. (2010). The water footprint: Water in the supply chain. Environmentalist.

[6] Egan M. (2011). The Water Footprint Assessment Manual. Setting the Global Standard. Soc. Environ. Account. J..

[7] Naranjo-Merino C., Ortíz-Rodriguez O., Villamizar-G R. (2017). Assessing Green and Blue Water Footprints in the Supply Chain of Cocoa Production: A Case Study in the Northeast of Colombia. Sustainability.

[8] Mekonnen M.M., Hoekstra A.Y. (2011). The green, blue and grey water footprint of crops and derived crop products. Hydrol. Earth Syst. Sci..

[9] Ewing B.R., Hawkins T.R., Wiedmann T.O., Galli A., Ercin A.E., Weinzettel J., Steen-Olsen K. (2012). Integrating ecological and water footprint accounting in a multi-regional input–output framework. Ecol. Indic..

[10] Hoekstra A.Y., Mekonnen M.M., Chapagain A.K., Mathews R.E., Richter B.D. (2012). Global Monthly Water Scarcity: Blue Water Footprints versus Blue Water Availability. PLoS ONE.

[11] Zhang G.P., Hoekstra A.Y., Mathews R.E. (2013). Water Footprint Assessment (WFA) for better water governance and sustainable development. Water Resour. Ind..

[12] Ercin A.E., Hoekstra A.Y. (2014). Water footprint scenarios for 2050: A global analysis. Environ. Int..

[13] Ogryczak W., Śliwiński T., Wierzbicki A. (2003). Fair resource allocation schemes and network dimensioning problems. J. Telecommun. Inf. Technol..

[14] Yager R.R. (2010). Lexicographic ordinal OWA aggregation of multiple criteria. Inf. Fusion.

[15] Liu G., Wang H., Qiu L. (2012). Construction of a Cooperation Allocation Initial Discharge Permits System for Industrial Source Points in a Lake Basin—A Case Study of the Taihu Lake Basin. Resour. Environ. Sin Yangtze Basin.

[16] Gini C. (1921). Measurement of Inequality of Income. J. Econ. Theory Econ..

[17] Chen X. (2004). Gini Coefficient and its Estimation. Statistical Research. Stat. Res..

[18] Zhou S., Du A., Bai M. (2015). Application of the environmental Gini coefficient in allocating water governance responsibilities: A case study in Taihu Lake Basin, China. Water Sci. Technol..

[19] Malakar K., Mishra T., Patwardhan A. (2017). Inequality in water supply in India: An assessment using the Gini and Theil indices. Environ. Dev. Sustain..

[20] Seekell D.A. (2011). Does the Global Trade of Virtual Water Reduce Inequality in Freshwater Resource Allocation?. Soci. Nat. Resour..

[21] Theil H. (1969). The Desired Political Entropy. Am. Polit. Sci. Rev..

[22] Soogwan D. (2009). The Impact of National and Local Development Policies on Regional Disparities in South Korea: 1985–2005. Asia Pac. J. Public Adm..

[23] Liberati P., Bellu L. (2006). Decomposition of Income Inequality by Income Sources. Kokugogaku Stud. Jpn. Lang..

[24] Clemente P.A., Alves A.D., Viana C.A.C., Guimarães A.M.H.N., Ferreira F.E. (2012). Socioeconomic indicators and oral health services in an underprivileged area of Brazil. Revista Panamericana Salud Publica (Online).

[25] Liu G., Shi L., Li K.W. (2018). Equitable Allocation of Blue and Green WaterFootprints Based on Land-Use Types: A Case Study of the Yangtze River Economic Belt. Sustainability.

[26] Liu J., Wu M., Yu Z. (2018). Evaluation of Environmental Impacts Due to Blue Water Consumption in China from Production and Consumption Perspectives. Int. J. Environ. Res. Public Health.

[27] Pute W.U., Wang Y., Zhao X., Sun S., Jin J. (2015). Spatiotemporal variation in water footprint of grain production in China. Front. Agric. Sci. Eng..

[28] Zhuo L., Mekonnen M.M., Hoekstra A.Y. (2016). The effect of inter-annual variability of consumption, production, trade and climate on crop-related green and blue water footprints and inter-regional virtual water trade: A study for China (1978–2008). Water Res..

[29] Hoekstra A.Y., Hung P.Q. (2002). A quantification of virtual water flows between nations in relation to international crop trade. Water Res..

[30] Wang L. (2005). Cooperative Water Resources Allocation among Competing Users. Ph.D. Thesis.

[31] Pi J.C. (2007). Leaders, followers and collective actions in communal cooperation: An extension based on the fairness-compatible constraint. China Econ. Q..

[32] Mookherjee D., Shorrocks A. (1982). A Decomposition Analysis of the Trend in UK Income Inequality. Econ. J..

[33] Xiao W., Qin D., Li W., Chu J. (2009). Model for Distribution of Water Pollutants in A Lake Basin Based on Environmental Gini Coefficient. Acta Sci. Circumst..

[34] Silber J. (1989). Factor Components, Population Subgroups and the Computation of the Gini Index of Inequality. Rev. Econ. Stat..

[35] Zou Z., Yun Y., Sun J. (2006). Entropy method for determination of weight of evaluating indicators in fuzzy synthetic evaluation for water quality assessment. J. Environ. Sci.-China.

[36] Xia J., Zhu Y. (2002). The measurement of water resources security: A study and challenge on water resources carrying capacity. J. Nat. Resour..

[37] Liu G., Wang H., Qiu L. A lexicographic quota model for allocating initial discharge permits for industrial source points in a lake basin: A case study for Lake Tai in Jiangsu, China. Proceedings of the IEEE International Conference on Systems, Man, and Cybernetics.

[38] Luss H. (1999). On equitable resource allocation problems: A lexicographic minimax approach. Oper. Res. Lett..

[39] Luss H., Smith D.R. (1986). Resource allocation among competing activities: A lexicographic minimax approach. Oper. Res. Lett..

